# Supplementary material for: The interactome of intact mitochondria by cross-linking mass spectrometry provides evidence for coexisting respiratory supercomplexes
Source: Mol Cell Proteomics. 2017 Dec 8;17(2):216–32. doi: 10.1074/mcp.RA117.000470 (PMC5795388; doi:10.1074/mcp.RA117.000470)
Supplement: Supplemental Data [file supp_RA117.000470_133922_0_supp_23978_fzffwf.zip › spectra_annotation/mito_spectra_annotation/466-1-3-1-17-1.pdf]

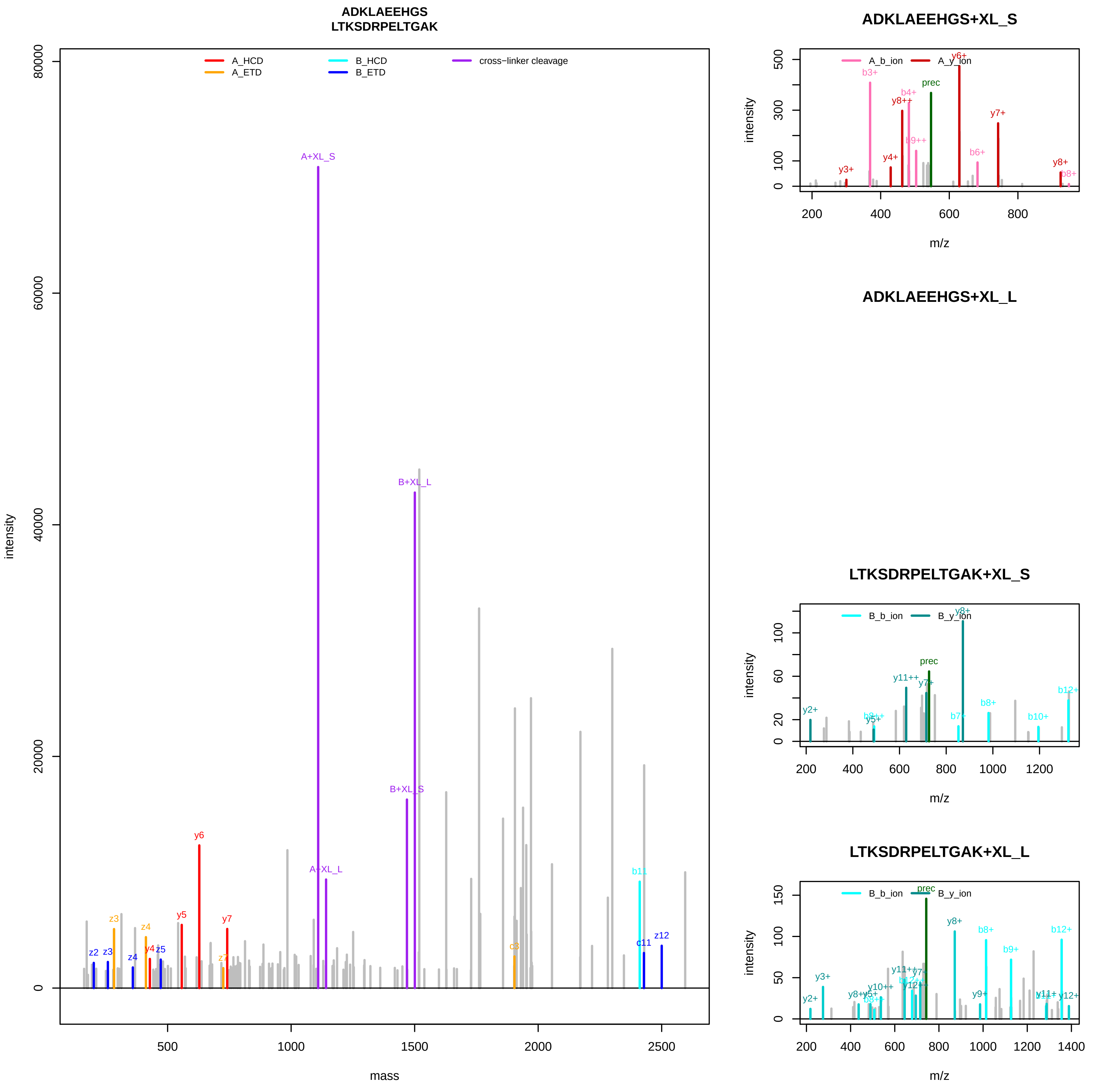
ADKLAEEHGS
LTKSDRPELTGAK
A_HCD
A_ETD
B_HCD
B_ETD
cross−linker cleavage
A+XL_S
B+XL_L
B+XL_S
A+XL_L
y6
y5
y7
y4
z2
z3
z4
z5
z3
z4
z7
c3
b11
c11
z12
intensity
mass
ADKLAEEHGS+XL_S
A_b_ion
A_y_ion
prec
b3+
b4+
y8++
y6+
y7+
b9++
b6+
y4+
y3+
y8+
b8+
m/z
ADKLAEEHGS+XL_L
LTKSDRPELTGAK+XL_S
B_b_ion
B_y_ion
y8+
prec
y11++
y7+
y2+
y5+
b7+
b8+
b10+
b12+
LTKSDRPELTGAK+XL_L
y3+
y2+
y8+
b8+
b9+
b12+
y11++
y10++
y9+
y11+
y12+
m/z
intensity
